# Observations on Ergot

**Published:** 1841-07

**Authors:** John B. Beck

**Affiliations:** Professor of Materia Medica and Medical Jurisprudence in the College of Physicians and Surgeons of New York


*Observations on Ergot:* By John B. Beck, M. D., Professor of Materia Medica and Medical Jurisprudence in the College of Physicians and Surgeons of New-York.

In the whole range of the materia medica there is no article more interesting in its effects on the human system than ergot. Given during labor, it possesses the curious property (possessed by no other substance that we know of,) of exciting uterine action, and facilitating in a most extraordinary manner, the whole process of delivery. Upwards of thirty years have now elapsed since its introduction into general practice in this country; and during portions of the same period, it has been extensively used in Great Britain and on the continent of Europe. After such ample experience, we should naturally suppose that every thing in relation to its action would be completely established. Such, however, is not the case. Several important points are still under dispute, and it is upon these that it is proposed to make a few observations in the following paper.

I. By some it is *denied that ergot possesses any such property as is generally ascribed to it.* On this point it would seem hardly necessary to say any thing. Whether ergot does or does not possess such property, is a question which must be decided by the observations and testimony of those who have used it, and the mass of recorded as well as unrecorded evidence which we possess on this subject, is so abundant, as one would suppose, would be sufficient to preclude all doubt.*

*Bayle has collated the reports of sixty-two authorities on the subject of ergot, and out of 1176 cases of lingering labor in which it was used, 1051 were more or less promptly terminated by it. In 111 cases, it failed to produce any effect, and in 14 the success was moderate.—*Bibliotheque Therapeutque, &c., par A. L. I. Bayle tome* iii, *p.* 534.

In addition to the foregoing mass of authority, I will only adduce the testimony of Dr. Ward of New-Jersey, who states that during six years he gave it to between sixty and seventy patients, and in every case, except one, it produced powerful uterine contractions in fifteen or twenty minutes after its administration.—*New-York Med. and Phys. Journal for* 1825, *vol.* iv, *p.* 212.

Notwithstanding all this, it is maintained by some high authorities that ergot does not act upon the uterus, and in support of this opinion it is alleged that it has been frequently given without any such effect having followed, and when it has taken place, it is explained upon the ground of its being a mere accidental coincidence, and that the uterine efforts would have been renewed just as certainly without its agency. Now that erogt has frequently been given without producing any effect on the uterus is readily admitted, and yet this by no means proves that it is destitute of the power ascribed to it. Some constitutions are doubtless not susceptible to its action. This we know to be the case with many agents, whose action on the human system is universally acknowledged. Besides, much of the alleged inefficacy of ergot may very readily be explained by the fact, now well known, that this article is not always precisely the same. From a variety of causes influencing the growth of this curious substance, independently of designed sophistications, it has been established that its properties differ very materially, and if these be not duly regarded, it is by no means wonderful that its use is frequently not followed by any effect. With regard to the supposition that the uterine action which follows its exhibition is a mere coincidence, it seems to me to be entirely done away with by the fact that the pains which are produced are entirely different in their character from those of ordinary labor. The latter are distinguished by perfect intermissions; while the former, are not only more severe, but they are continuous until the labor is completed. Females themselves are perfectly conscious of this difference in the two kinds of pain, and by them this difference has been frequently described. Besides this, the uniformity and rapidity with which the pains come on after the exhibition of ergot, is altogether irreconcilable with the supposition of its being a mere coincidence. If the pains came on at remote and variable periods, then indeed might there be some ground for denying the agency of ergot in producing them. This, however, is not the case. As a general rule in from five to twenty minutes, severe and forcing pains come on,* and after continuing for an hour or more, if the de-

*By Dr. Prescott the time was precisely marked in twenty cases. "In two of these, the increased strength of the pains and the continued action commenced in seven minutes from the time the decoction was taken; in one case, it was eight minutes; in seven, it was ten; in three, eleven; and in three others, it was fifteen minutes; in the four remaining cases there was no apparent operation until twenty minutes had expired."—*A Dissertation on Ergot, by Oliver Prescott, A. M., p.* 11, *Boston*, 1813.

Dr. Ward, as already quoted, states that he used it in sixty or seventy livery be not completed, the same effects may be reproduced by a repetition of the dose. Now, if all this does not prove that the ergot is the cause of the uterine action, I am at a loss to conceive what kind of evidence will establish the action of any medicinal agent on the human system. If we still doubt in relation to ergot, we may with equal propriety doubt concerning the operation of ipecacuanha on the stomach, or of calomel on the liver. Although there can therefore be no reasonable question about the operation of ergot, yet it is certain that it sometimes fails. This is a fact which has been frequently noticed by those who have prescribed it. Professor Dewees states that he has "in several instances failed to produce the slightest effect with the ergot procured at one shop, whilst that from another, in the same patient, has been as prompt as it was efficacious."* The same thing has been observed by others. In a large majority of cases this can easily be accounted for. The character of this article is modified by a number of circumstances, all of which should be attended to if we wish to have it genuine. As these are important in a practical point of view, they are deserving of the greatest attention.

In the first place, the character of the season, as to dryness or moisture, appears to influence very materially the quality of the ergot. According to Burnett, it has been ascertained that the principle of the ergot resides in the diffluent peridium or external covering. Now if heavy rains fall at the time when the peridium is soft and moist, it will be washed away and the hardened nucleus, if wholly denuded, will be utterly inert. If the weather be fine during the season of maturation of the fungus, the diffluent peridium will be dried upon the spur, and the ergot be in its most active state. Hence it is, that although moisture favors the early growth of the ergot in the spring and summer, it requires a dry autumn to ensure its activity.†

---

cases, and in all excepting one case, it produced "powerful uterine contraction in fifteen or twenty minutes after its administration."—*N. York Med. and Phys. Journal, vol.* ii *p.* 212.

Mr. John Paterson of Aberdeen, states that he used it in eight cases, and it acted strongly in all in less than five minutes after it was administered.—*Edin. Med. and Surg. Journal for Jan.* 1840, *p*, 142.

*American Journal of Medical Sciences, vol. i, p. 255.

†Outlines of Botany, by Gilbert Burnett, Prof. of Botany in King's College, London, p. 207, Upon the same principle he explains the fact, that the grain in which the spur prevails in equal proportions, will in some years produce the dry gangrene, while in others it will not.

In the second place, the period when it is gathered has an influence on the character of the ergot. According to the experiments of Dr. Kluge of Mendelurtz, it would seem that it only displays its active properties, when collected before the cutting of the parent crop. At the Maternite of Mendelurtz, trials were made upon fifteen females, and the result was, that what was gathered before harvest was very energetic, while that collected after harvest was altogether powerless.* Whether this be true in its full extent or not, certain it is that there is a great difference in the strength of the article, according to the time when it is gathered. A recent writer, Dr. Green, who states that he used it in nearly one hundred cases, says that when gathered from the standing grain, and about the period when it is ripe for the harvest, he has found it not only more certain in its action, but "in doses of eight or ten grains, to prove as efficient in increasing the uterine powers, as the scruple or half drachm doses of the ones obtained in the ordinary way."†

In the third place, the time it has been kept modifies the quality of the ergot. Although some experiments of Lorinser would seem to show that so far as its action on the stomach is concerned, it retains its active properties for two years,‡ yet the result of general observation has shown that its influence over the uterus is impaired if it be kept over the year in which it is collected. Like all other vegetables too, it is easily acted on by heat and moisture. To have it good, therefore, it should be fresh; and it ought to be kept in bottles tightly stopped, and it should not be pulverized until required for use.

In the fourth place, a fictitious ergot has sometimes been sold for the real article. In this country and on the continent of Europe, where rye is extensively cultivated, and where of course there is an abundance of ergot, this is a fraud which is not likely to be met with. In England, however, where much less rye is grown, the ergot is occasionally very scarce, and this has led to a variety of impositions. Dr. O'Shaughnessy of London states, that a specimen of suspected ergot was once given to him for analysis, and he found it to be composed of the sulphate of lime, which had been cast in a mould and colored, so as to imitate very closely the natural ergot.§ Mr. Wright says he has several times observed the

---

*American Journal of Medical Sciences, vol. xii. p. 515.
†New-York Journal of Medicine and Surgery, for Jan. 1841. p. 23.
‡Edin. Med. and Surg. Journal for 1826, p. 453.
§Lancet for 1830–31, vol. i. p. 638.

ergot to be adulterated with common paste; "a fraud," he suspects, "of very frequent occurrence, though not of very easy detection; for the process of baking generally modifies the starch, so that it can scarcely be indicated by iodine."*

The foregoing causes appear to me abundantly sufficient to account for the discrepancies in the statements which have appeared in relation to the action of this singular substance, as well as for the occasional failure which attends its use.

II. *Acting thus powerfully on the uterus, does ergot produce any effect upon the child?* This is a question of great interest, and one which involves consequences of great importance, not merely in a professional, but in a moral point of view. On this subject, the opinion of the profession is divided. While some maintain that it produces no effect, at least no injurious effect upon the child; others contend that it frequently proves destructive of life, and that the general use of it is one of the causes of the great increase in the number of still-born children. An attentive examination of the subject, in all its bearings, will, I fear, but too certainly lead to a conclusion in favor of the latter opinion. From the peculiar effect of the ergot upon the uterus, it is evident that the child must sustain a degree of pressure entirely different from what it does in ordinary labor. In the first place, it is much greater. In the second place, it is unremitting and continued, and that too for a considerable length of time. Now, it is by no means unreasonable to suppose that this pressure may frequently prove injurious and even fatal to the child. This would be more especially likely to happen in cases where the waters are discharged early, and where the uterus is contracting directly upon the child. In natural labor, the child has time to recover from the effects of pressure during the intervals between the pains, while here no such chance is afforded. And it is not irrational to suppose that the design in making the pains of labor intermitting, was not merely to allow the mother time to recover her strength, but also to enable the child to recover from the effects of pressure. That continued pressure may and does prove injurious to the child even in cases of ordinary labor, where this process is protracted, either from the disproportionate size of the child, or from the resistance of the parts through which it is to pass, is a fact well known. How much more likely is this to happen where an unnatural and unremitting pressure is kept up, as is the case under the influence of ergot? From these general considerations it would seem not merely perfectly natural but unavoidable that in

*Edinburgh Med. and Surg. Journal, Oct. 1839, p. 297.

many cases, the child must suffer from the use of ergot. After all, however, this is a question which must be decided by facts, and these will tend still further to countenance this opinion. So early as the year 1812, it was suggested by the editors of the New-England Journal of Medicine and Surgery, that while fully convinced of the parturient powers of the ergot, they were apprehensive that an evil of great magnitude not unfrequently resulted from its use; and that was the death of the child. They stated that they had been led to this apprehension from "observing that in a large proportion of cases where the ergot was employed, the children did not respire for an unusual length of time after the birth, and in several cases the children were irrecoverably dead."* Since then a large amount of testimony has been furnished confirmatory of the truth of this suggestion. In the same Journal,† a case is recorded of a female in her third labor, who was delivered of twins. After the first child was born, which was living, an hour elapsed without the recurrence of a single pain, in consequence of which it was determined to administer the ergot. Fifteen grains in powder were accordingly given in a little water. In fifteen or twenty minutes the pains came on and continued without remission till the child was born, which was in about twenty minutes from the time the pains commenced, the head being born first as in natural labor. The child, however, was still-born, and every effort to resuscitate it failed. It was in every respect as fine a child as the first, perfectly fresh and firm. The writer remarks that "every one who is acquainted with the facility with which, in a case of twins, the second child makes its way into the world, will consider the death of the child in this instance as an unusual occurrence."

Dr. Ward of New-Jersey, whose experience with this article appears to have been extensive, and who speaks of it as a valuable agent in many cases, nevertheless admits the danger which attends the child from its use. "In all the cases," he says, "in which I have given it, unless the child was expelled very soon after the powerful contractions came on, it suffered very much, and would lie for some time without breathing." Again he says, "from my own observations, with regard to the ergot, as well as from other correct sources of information, I am led to conclude that in most cases, after giving it, unless the child is expelled in forty minutes after the powerful contractions come on, it will be born dead."‡

*Vol. i, p. 70.

†Vol. ii, p. 353.

‡New-York Med. and Phys. Journal, vol. iv, p. 212.

The late Dr. William Moore, a veteran practitioner of obstetrics in this city, after detailing some cases, gives his opinion in relation to ergot in the following terms: "It appears to be injurious to the child at all times; for in every case in which I have seen it exhibited, the child has been still-born and in the greater part of them it was not possible to restore it to life.*

Dr. Hosack states that he gave the ergot in three cases, and "although no evidence existed previous to the use of the medicine, that the fœtus was not living, in every case in which it was administered, the child was still-born."†

Dr. Chatard, of Baltimore, made two reports in relation to the effects of ergot. In the first, out of twelve cases in which it was given, six of the children were still-born.‡ In a second report, out of twenty-five cases, eight were still-born, two of whom were however resuscitated.§

Dr. Holcombe, of New-Jersey, says, "more children, I am satisfied from what I have seen and heard, have already perished by the injudicious use of ergot, during the few years which have followed its introduction into the practice of this country, than have been sacrificed by the unwarrantable use of the crotchet for a century past."||

Dr. Church, in seven cases, which he details, in which the ergot was used, had five children still-born. Although he thinks that in these cases the ergot had nothing to do with this result, yet he confesses that he "has no doubt if given in cases where there is great rigidity of muscular fibre, before the labor is advanced, when the os uteri is undilated, the external parts unrelaxed, and when blood-letting has not been premised, that the powerful and continued efforts of the uterus, caused by the ergot, will prevent the retreat of the child's head after it has advanced within the bones, and that the unceasing pressure may in some instances occasion death."**

Dr. Davies, of London, reports ten cases in which the ergot was used. In four, the child was still-born. In a fifth, the child was apparently still-born, but soon recovered. In all the still-born cases, it appears that the child was not delivered until upwards of an hour had elapsed after the administration

---

*Compendium of Midwifery, by Samuel Bard, M. D., p. 214, 4th ed.
†New-York Med. and Surg. Journal, vol. i. p. 205.
‡New-York medical Repository, vol. xx. p. 17.
§Ibid, vol. xxi. p. 160.
||Philadelphia Journal of the Med. and Phys. Sciences, vol. xi. p. 318.
**Philadelphia Journal of Med. and Phys. Sciences, vol. viii. p. 139.

of the ergot. In the first, two hours elapsed; in the second, a little more than an hour; in the third, six hours; in the fourth, a little over an hour.*

Mr. T. Chavasse, of Birmingham, states that in eighteen cases in which the ergot was used, the children were still-born.†

Mr. Jukes, of Birmingham, says that out of six cases in which he used it, five of the children were still-born.‡

Mr. P. H. Chavasse reports nine cases in which its use was followed by the birth of still-born children, and in all before he administered the ergot, " there was every indication of the children being alive."§

Mr. Elkington says that "several of his patients who took it, had still-born children."||

Mr. John Paterson, of Aberdeen, used the ergot in eight cases, and in three, the children were still-born—" than which," he says, "no stronger evidence need be adduced of its extreme danger." In the three cases alluded to, he states, that he satisfied himself before its administration that the children were not only alive, but apparently strong and healthy; but as soon as the action of the medicine commenced, these impressions became gradually less sensible to himself as well as the mother. And he adds his doubts whether by the use of this article more deaths are not occasioned than by the use of instruments.**

In addition to the foregoing, I adduce the following communication from one of my professional friends in this city, whose long experience entitles his opinions in relation to practical matters, to the highest consideration.

*New-York, January 14th*, 1841.

*My dear sir:*

After what I considered a fair and full trial, I formed an opinion on the use of ergot, twenty-five years ago, and one which has governed me in practice ever since. I consider it a valuable article of the materia medica, to be used with great caution and only in cases of clear necessity. I have reasons satisfactory to my own mind for believing that it has frequently destroyed fœtuses and produced sterility in mothers. Entertaining this opinion I am surprised to see by some late publications that this article continues to be resorted to by some

---

*New-England Journal of Med. and Surgery, vol. xv. p. 18.

†Transactions of the Provincial Med. and Surg. Association, vol. iv. copied into the Transactions of the N. Y. State Med. Society, vol. iii. p. 353.

‡Ibid, vol. iii. p. 354. §Ibid, vol. iii. p. 355. ||Ibid, vol. iii. p. 354.

**Edinb. Med. and Surg. Journal for January 1840, p. 142.

practitioners under very trivial pretexts. I mean on occasions where, to say the least, it is totally unnecessary. It hastens labor, it is true, but I entertain so high a respect for the intelligence of nature, that I consider this hazardous method of bringing a child *into* the world before its time, as little better than sending it *out* before its time.

Yours truly,
CYRUS PERKINS.

Prof. J. B. Beck.

The facts which have thus been detailed, would seem to be abundant to show that the use of this article has in many cases proved injurious to the child. That it does not prove so in all cases, and that in the hands of those who have used it prudently and judiciously it has never produced such an effect, is certainly no argument against the correctness of this conclusion. Even by those who have most frequently observed its fatal effects upon the child, it is admitted that this does not uniformly take place. The circumstances under which this difference of effect occurs, are easily explicable. As the danger to the child appears to be owing to the degree and duration of the pressure to which it is subjected, it would seem evident that just in proportion as the uterine organs are in a condition to admit of a speedy delivery after the ergot begins to operate, will the danger of the child be lessened; and on the other hand, in proportion as the delivery is protracted will the danger be increased. This corresponds with the observation of Dr. Ward, already quoted, that whenever the child is not delivered in forty minutes after the action of the ergot commenced, it is generally still-born. For the same reason too it has been found more injurious when used in first labors than in subsequent ones.

III. *Is ergot capable of producing any effect on the uterus anterior to the full term of gestation?* On this point there is also a great difference of opinion. Some contending that it acts only at the full period and when the process of labor has already commenced; while others assert that it exerts its influence at any period of pregnancy. To settle this question a great number of experiments have been made upon animals, the result of which is, that while in the majority it produced no effect, yet in a number it succeeded.

By Dr. Erskine, several experiments were made upon cats, at various periods of Pregnancy, and in every instance it is stated that he succeeded in producing abortion.*

*Philadelphia Journal of Med. and Phys. Sciences, vol. xi. p. 112.

By Dr. Oslere, experiments were also made upon animals, and with similar results. The first was on a sow, who was supposed to be in her seventh week of pregnancy. One drachm of the ergot was given and repeated again in the course of three hours. In the course of the night she had aborted nine small pigs, about the size of common mice. His second experiment was upon a cow, which was supposed to be with calf, though not sufficiently advanced to be certain of the fact. Two ounces of ergot in powder were given about ten o'clock in the morning, and after suffering severe pain she aborted at six o'clock in the evening of the same day. The abortion was about the size of a common full grown rat, but very imperfectly formed. His third experiment was on a cat that appeared to be near her time of delivery. Sixteeen grains of ergot in powder, mixed with butter, were given at eight o'clock in the evening, and the animal confined in a room. On visiting her the next morning, she was found to have been delivered of four kittens, all of which died during the day.*

Dr. Oslere states as the result of his experiments, that he has not the least hesitation in believing that the ergot is capable of producing abortion at any period of utero-gestation.

Dr. Chatard, of Baltimore, tried its effects upon six cats, all more or less advanced in pregnancy. On the first, it acted as an emetic; the second was slightly purged; the third, fourth, and fifth, were not at all affected by it, although the last took a double dose of it at once, i. e. two drachms in powder; the sixth, half advanced in pregnancy, to which he gave but one drachm, had her legs paralyzed for a short time, in less than one hour, and abortion took place in twenty-four hours, preceded by considerable hemorrhage.†

More recently several experiments were made upon different animals, by Mr. Wright, without producing any effect.‡

The only inference to be drawn from the foregoing facts is, that although ergot is capable of causing abortion in animals, it does not do so with any degree of certainty or uniformity.

That ergot has the power of producing premature labor in the human subject, is now established by such a number of well attested cases, as to leave no reasonable doubt on the subject.

Dr. Oslere, in a paper published in 1825, states that this had been successfully practised by Prof. James, in the case of

---

*Philadelphia Journal of Med. and Phys. Sciences, vol. xi. p. 113.

†New-York Medical Repository, vol. xxi. p. 163.

‡Edinb. Med. and Surg. Journal for Jan. 1840, p. 31.

a woman whose pelvis was too small to permit the passage of a full grown child. She had several times been pregnant, and in every case the operation of embryulcia had been resorted to for her delivery. In a subsequent pregnancy, Dr. James suggested the propriety of bringing on premature labor by the use of ergot, and this was accomplished with success, not only in this but in several subsequent pregnancies.*

Dr Chatard relates a case in which hemorrhage occurred at the fourth month of pregnancy. Every means used to arrest it having failed, twelve grains of ergot were given with the effect of arresting the hemorrhage. On the next day, the hemorrhage returning, a similar dose was given, and in a few hours the female aborted.†

Dr. Dewees relates the following case: A female, whose husband had been absent a long time, became pregnant by illicit connexion. Wishing to conceal her guilt she applied to a physician, who gave her some powders which he said would produce abortion. After taking several of them, severe pains came on, with hemorrhage. In this state she was found by Dr. Dewees, and shortly after she was delivered of twins at about the fifth month. On examining a powder which was left, and which was similar to those she had taken, Dr. Dewees found it to be a drachm of powdered ergot.‡

Dr. Weike relates the case of a female attacked with hemorrhage in the fourth month of pregnancy. After continuing violently for three days without any appearance of the expulsion of the fœtus, and when the woman was apparently dying from the loss of blood, eight grains of ergot were ordered to be given every half hour. After the third dose labor pains came on, and she had scarcely taken the fourth before the fœtus was expelled.§

The most satisfactory testimony, however, on this subject, is that which has been furnished within a few years by several British physicians. In 1834 Dr. F. H. Ramsbotham, of London, detailed six cases, in which it was necessary to induce premature labor, and in all it was successfully brought about by the use of ergot. In the first case the pregnancy had advanced to eight months; in the second to seven and a half; in the third to seven and a half; in the fourth to seven and a half; in the fifth to eight; and in the sixth to seven and a half months.||

---

*Philad. Journal of the Med. & Phys. Sciences, vol. xi. p. 114.

†N. Y. Med. Repository, vol. xxi. p. 163.

‡American Jour. Med, Sciences, vol. iii. p. 408.

§British and Foreign Review, vol. ii. p. 276.

||London Medical Gazette, Jnne, 1834, p. 436.

In a subsequent paper, Dr. Ramsbotham has given an account of his practice in those cases in which, from the narrowness of the pelvis, he was obliged to resort to the induction of premature labor. Of these he states that in all he had had sixty-two cases. In thirty-six cases the membranes were punctured, and in twenty-one of these the children were born alive, and sixteen were still-born. In twenty-six the labor was induced by ergot, without any other means being used; of these twelve were born alive, and fourteen still-born. Besides establishing, beyond all doubt, the fact that ergot is capable of exciting the uterus into action anterior to the full term, this report is important in another respect, and this is particularly noticed by the author. It is that the number of still-births in these cases was much greater in proportion in those in which the ergot was used, than in those in which the practice of puncturing the membranes was resorted to. Dr. Ramsbotham adds the remark, that he has seen the stimulating effects of ergot on the uterus in numerous cases of dangerous hemorrhage in the early months, when it was desirable to procure a complete evacuation of that organ, and where no manual or instrumental means could be put in practice.*

Dr. Paterson, of Glasgow, has reported the case of a woman in whom he succeeded in bringing on premature labor in two successive pregnancies by the use of ergot. In both cases it was about the seventh month.†

A similar case is reported by Dr. Heane, in which the ergot effected a premature delivery at the seventh month.‡

Another case occurred under the care of M. Dubois, of Paris, in the person of a dwarf, who in her first pregnancy was obliged to be delivered by perforating the head, and thus bringing away the child. On becoming pregnant a second time, he determined on bringing on premature labor at the eighth month, by dilating the os uteri and the use of ergot. This was accordingly done with success, and a living child delivered.§

The foregoing evidence is conclusive as to the fact that ergot does exert its action on the uterus anterior to the full term of pregnancy. What the earliest period is, at which it is capable of producing this effect, it is impossible at present to determine.

IV. *To what extent are we justified in using ergot?* If

---

*London Medical Gazette for June, 1839, p. 422.

†Ibid, for June, 1839, p. 333.

‡Ibid, for January, 1839, p. 639.

§Dunglison's American Med. Intelligencer, vol. iv. p. 126.

there be any truth or force in what has been said in relation to the effects of this article on the child, the answer to this question is obvious. In a professional as well as moral point of view, we have no more right to trifle with the life of the child than we have with the life of the mother. When, however, from the nature of the case, it becomes manifest that the life of the mother is in danger, we are not merely justified in using, but it is a positive duty to do so, every means to save her, disregarding every consequence that may result to the child. Now it is for such contingencies that I conceive that ergot ought to be reserved. It should accordingly, I think, never be used except in cases where nature is incompetent to a safe delivery. By too many, it is to be feared, it has been and still is used merely as a *time-saving* agent. Than this, I cannot conceive of any practice more unjustifiable and reprehensible. As a general rule, nature is competent to a safe delivery, and we may rest assured that the best plan is to leave her alone to accomplish the work. Artificial and violent interference, whether it be applied in the shape of instruments or by the use of ergot, cannot but be improper.—*Transactions of the Med. Soc. of New-York. Vol. V. Part* 1.

